# Preterm Hypoxic–Ischemic Encephalopathy

**DOI:** 10.3389/fped.2016.00114

**Published:** 2016-10-20

**Authors:** Krishna Revanna Gopagondanahalli, Jingang Li, Michael C. Fahey, Rod W. Hunt, Graham Jenkin, Suzanne L. Miller, Atul Malhotra

**Affiliations:** ^1^Monash Children’s Hospital, Melbourne, VIC, Australia; ^2^The Ritchie Centre, Hudson Institute of Medical Research, Melbourne, VIC, Australia; ^3^Department of Paediatrics, Monash University, Melbourne, VIC, Australia; ^4^The Royal Children’s Hospital, Melbourne, VIC, Australia; ^5^Murdoch Childrens Research Institute, Melbourne, VIC, Australia; ^6^Department of Obstetrics and Gynaecology, Monash University, Melbourne, VIC, Australia

**Keywords:** cerebral palsy, asphyxia, preterm brain injury, excitotoxicity, encephalopathy

## Abstract

Hypoxic–ischemic encephalopathy (HIE) is a recognizable and defined clinical syndrome in term infants that results from a severe or prolonged hypoxic–ischemic episode before or during birth. However, in the preterm infant, defining hypoxic–ischemic injury (HII), its clinical course, monitoring, and outcomes remains complex. Few studies examine preterm HIE, and these are heterogeneous, with variable inclusion criteria and outcomes reported. We examine the available evidence that implies that the incidence of hypoxic–ischemic insult in preterm infants is probably higher than recognized and follows a more complex clinical course, with higher rates of adverse neurological outcomes, compared to term infants. This review aims to elucidate the causes and consequences of preterm hypoxia–ischemia, the subsequent clinical encephalopathy syndrome, diagnostic tools, and outcomes. Finally, we suggest a uniform definition for preterm HIE that may help in identifying infants most at risk of adverse outcomes and amenable to neuroprotective therapies.

## Introduction

Worldwide, 11.1% of all live births every year are preterm (born before 37 weeks’ of completed gestation) (1), and the rate appears to be rising (2). In high-income settings, advances in neonatal care for preterm babies have greatly increased survival rates; however, premature babies remain at risk of serious health problems, including respiratory distress syndrome, bronchopulmonary dysplasia, retinopathy of prematurity, feeding difficulties, necrotizing enterocolitis, infections, longer hospital stays, and adverse long-term outcomes. In low-income countries, prematurity is a leading cause of neonatal and infant mortality (3).

Infants born prematurely have a high incidence of neonatal brain injury, with detrimental effects on motor, cognitive, behavioral, social, attentional, and sensory outcomes. Increased survival in lower gestational ages is accompanied by increased suboptimal neurodevelopmental outcomes (4–9). The incidence of any adverse neurodevelopmental outcomes varies with up to 17% of preterm infants described as having major impairments and, for babies weighing less than 1000 g at birth, up to 42% of survivors having minor impairment. The incidence of cerebral palsy (CP) and other adverse neurodevelopmental outcomes increases with decreasing gestational age at birth [from 5 to 10% in very low birth weight infants (<1500 g), 6 to 20% in extremely premature babies (<26 weeks’ gestation), and up to 25% in those born at a gestational age of less than 25 weeks] (10).

Historically, the best described neuropathological correlates of “encephalopathy of prematurity” (11) have been periventricular leukomalacia (PVL) with associated axonal/neuronal disruption and severe germinal matrix/intraventricular hemorrhage, with or without, posthemorrhagic ventricular dilatation (12). More recently, abnormalities of white matter (WM), with disrupted development of other cerebral structures (such as hippocampus, basal ganglia, corpus callosum) have been described in the context of premature brain abnormalities observed in the neonatal period (13, 14).

Preterm cerebral hypoxic–ischemic injury (HII) may occur rarely because of a recognized sentinel event. However, in the setting of coexistent factors, such as infection, inflammation, growth restriction, severe hypoglycemia, or hyperoxia, the contribution of individual pathologies may be difficult. For these reasons, perinatal HII to the premature brain, its clinical manifestations, recognition, and monitoring has not been well studied. In contrast, the diagnosis of HIE in full-term infants is aided by defined objective criteria involving perinatal factors, such as acidosis, Apgar scores, and the need for resuscitation, with standardized neurological examination and neurodevelopmental outcomes (15–17).

In this review, we highlight the contribution of preterm HIE to the injury complex in the developing brain. We propose an algorithm for a universal definition of preterm HIE, based on recognition of the specific manifestations of a perinatal HII. An accurate and uniform definition of preterm HIE may help to identify a homogeneous population of infants, who may be eligible for future clinical intervention trials.

## Premature Brain Development and Pathophysiology of Hypoxic–Ischemic Insult

**Figure 1:**
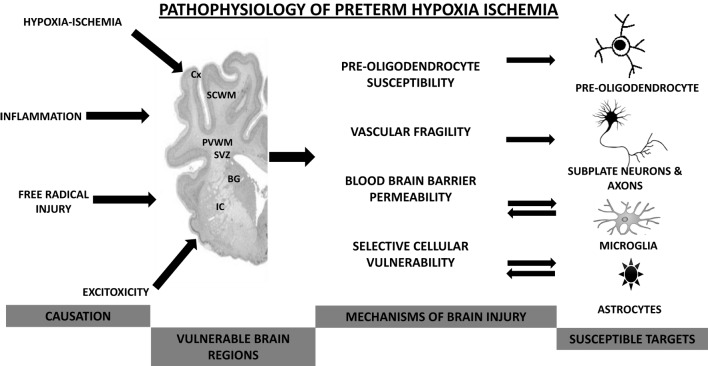
**Pathophysiological mechanisms involved in preterm hypoxic–ischemic encephalopathy**. Cx, cortex; SCWM, subcortical white matter; PVWM, periventricular white matter; BG, basal ganglia; IC, internal capsule.

Between 24 and 40 weeks of gestation, the human brain undergoes rapid changes that make the developing brain vulnerable to injury from hypoxia–ischemia, inflammation, free radical, and excitotoxic damage (Figure 1). There is growing understanding of the etiology of preterm brain injury, involving interactions between an immature brain and vulnerable WM developmental processes (18). Key developmental processes during this time include the *development of cerebral WM, proliferation zones, and neuronal structures. WM development* involves pre-myelinating oligodendrocytes (OLs), axons, microglia, and neurons (subplate and late migrating GABAergic neurons). Pre-OLs are the predominant cell lineage present in human cerebral WM from 24 to 40 weeks of gestation that differentiate into myelin-producing OLs. These mature forms of OLs do not become abundant until after term, and tolerate hypoxic insult better than pre-OLs. Microglial cells, which become abundant during the third trimester, are capable of generating inflammatory cytokines, enhancing cytotoxicity and generating free radicals when exposed to hypoxia and infection. Subplate neurons are a collection of neurons located beneath the cortical plate, reaching their peak mass and developmental impact by 24–32 weeks of gestation. Development of the subplate is closely linked with development of cerebral cortex, deep nuclear structures (especially thalamus and formation of area-specific thalamocortical connections), and axons. The second significant developmental difference is the presence of *two proliferative zones* [dorsal cerebral subventricular zone (SVZ) and the ventral germinative epithelium of the ganglionic eminence]. The fetal SVZ is directly related to the evolutionary expansion of the human cerebral cortex. The third key process is the *development of neuronal structures*, such as thalamus, cerebral cortex, and basal ganglia (11, 12, 18, 19).

Even without exacerbating factors, preterm birth is associated with subtle WM pathology (20). One theory is that the OL precursors and subplate neurons are exquisitely sensitive to pro-inflammatory cytokines, hypoxia, and oxidative stress (21). The principle pathogenic mechanism underlying neurological damage in HIE resulting from hypoxemia, ischemia, or both, is deprivation of glucose, and oxygen supply that causes a primary energy failure and initiates a cascade of biochemical events leading to cell dysfunction and ultimately to cell death (22–24).

The phases of cell death and biochemical interactions at cellular level in HII are well described and studied. *Primary energy failure*, where depletion of oxygen prevents oxidative phosphorylation, disrupting Na–K pump activity is followed by anaerobic metabolism with accumulation of lactic acid (25). The failure of the transmembrane Na–K pump results in the intracellular accumulation of sodium, calcium, and water (cytotoxic edema) leading to membrane depolarization, excessive release of excitatory neurotransmitters (particularly glutamate), increase in the intracellular concentration of calcium, activation of phospholipase, and generation of free radicals. The release of glutamate results in further calcium accumulation and sodium retention contributing to cell damage (26–28). *Excitotoxicity* refers to cell death mediated by excessive stimulation of excitatory amino acid receptors in response to dicarboxylic acid glutamate and, at the cellular level, is believed to be fundamental in hypoxic–ischemic damage to neurons (28). With the restoration of blood flow, there is a brief period of normalization of cerebral metabolism called a latent period. The latent period is believed to vary depending on the severity of the hypoxic–ischemic insult. The more severe the insult, the shorter is the recovery period (29–31). The *secondary energy failure* phase begins 6–48 h after the initial insult. The exact mechanisms of secondary energy failure are unclear but are attributed to oxidative stress, excitotoxicity, and inflammation (32, 33) and ultimately results in cell death through either apoptosis or necrosis, depending on the region of the brain affected and the severity of the insult. Necrosis dominates in severe injury, whereas apoptosis is observed in milder insults (10, 34). Following HII, a cell may undergo nitric oxide-mediated necrosis, when endogenous inhibitors of apoptosis are abundant, or apoptosis, when the inhibitors are deficient (35, 36). Mitochondrial dysfunction appears to play a crucial role in determining whether the cells affected by hypoxia–ischemia undergo necrosis or apoptosis (37, 38). Apoptosis may be predominant in the premature brain through upregulation of the key elements, such as caspase-3, caspase-12, and BAX (39, 40).

## Hypoxic Ischemia in Preterm Population

The pathophysiology of HII in the premature brain is particularly complex. How HI damage affects the developing brain is determined by the severity, intensity, timing of asphyxia, in addition to selective cellular vulnerability, and immaturity of the brain (36, 41). The following summarizes the distinguishing effect of HII on key components of the developing brain and the susceptibility of the immature brain to the effects of HII.

### Vascular Fragility

The thin, delicate vessels of the developing brain may not sustain effective blood flow to compensate for HII because of the underdeveloped distal arterial network and an immature cerebral auto regulatory capacity (12). The peripheral arteries in the growing brain lack collateral vessels and have limited vasodilatory function in response to HII, making them more susceptible to hypoxic injury (42).

### Blood–Brain Barrier Function

The effect of hypoxia on the blood–brain barrier function in the developing brain can be profound. HII results in altered function and increased permeability of the BBB. The hypoxic insult affects the important cellular and functional components of the BBB, the astrocytes, the tight junctions of endothelial cells, and the pericytes (43, 44).

### Developing Neuroglial Cells

Astrocytes are the predominant cell population in the CNS. They provide structural and metabolic support; they play a crucial role in scavenging high levels of excitatory neurotransmitters and are an important constituent of the BBB (6). In response to HII, astrocytes influence neuronal survival. Astrocytes play a neuroprotective role following insult, by promoting erythropoiesis (45); however, sustained HII can lead to a decreased functioning astrocyte population and, thereby, greatly decrease neuronal regeneration (46).

### Pre-Oligodendrocytes and the Immature Oligodendrocytes

Oligodendroglial maturation involves four sequential stages, the oligodendroglial progenitor, the pre-OL (or late oligodendroglial progenitor), the immature OL, and the mature myelin-producing OL (12). The late oligodendroglial progenitors predominate in cerebral WM and SVZ and, at 28 weeks of gestation, account for 90% of the total OL population (47). Pre-OLs and OL progenitors are highly susceptible to hypoxia, and this vulnerability is central to the pathogenesis of preterm WM injury and PVL (48, 49). The mechanism of damage to OLs results from release of excitatory amino acids (glutamate, GABA, and aspartate) cytokine and free radical-mediated injuries, and the inflammatory response induced by HII (12, 49–52).

### Selective Vulnerability

In the developing brain, certain regions and cells appear vulnerable depending on timing and the severity of the insult. In the preterm brain, the subplate neurons and OL precursors are most vulnerable, and in the term brain, projection neurons especially in the deep gray nuclei are at greatest risk during ischemic insults. Subplate neurons are the earliest and the most transient cell population of the neocortex – and are affected by HI (53). Loss of these cells results in abnormal thalamocortical connectivity and may explain the visual and somatosensory impairment seen in prematurely born infants suffering perinatal HII (15, 54). Several studies have shown that the late OL progenitors appear to be the most vulnerable in this lineage, and they neither mature nor develop following injury. The selective vulnerability could result from expression of the receptor subtypes that favor calcium entry and excitability and inefficient endogenous antioxidant mechanisms (48, 55, 56).

## Preterm Hypoxic–Ischemic Encephalopathy – Definitions, Incidence, Clinical Spectrum, and Recognition

Definitions used to identify preterm HIE vary across available studies. Chalak et al. (57) screened preterm babies (33–35 weeks) using NICHD criteria for hypothermia, whereas Logitharajah et al. (58) included Apgar scores of less than 5 and 7 at 1 and 5 min, cord pH <7, sentinel event, and need for resuscitation. Schmidt and Walsh (59) included babies with 5-min Apgar score less than 6, cord or initial pH <7, base deficit >15 mmol/L, sentinel event, and clinical evidence of encephalopathy, and the severity of HIE was based on modified Sarnat HIE staging.

## Incidence/Epidemiology

There is a paucity of literature on the true incidence and epidemiology of HIE in infants born preterm. Depending on the definition used, the reported incidence across studies varies from 1.3/1000 live births to 5–9/1000 live births (57, 59, 60). The gestational age for inclusion also varies slightly across studies. In a study by Salhab and Perlman (60), most babies were close to 34 weeks (range 31–36), whereas Chalak et al. (57) and Schmidt and Walsh (59) reported outcomes on infants between 32 and 35 weeks. The major limitations in these studies are small sample sizes, retrospective examination, and variable inclusion criteria used to identify the babies with hypoxic ischemia. The Salhab study included babies with umbilical cord pH <7, whereas the Schmidt study screened infants of 32–36 weeks of gestation for HIE on the basis of a 5-min Apgar score less than 6. In a study by Logitharajah et al. (58), up to 30% of babies with preterm HIE had a cord pH >7, so only including infants with a pH <7 might underestimate preterm HIE. Most of the studies quoted above had an incidence for moderate to severe HIE in a preterm population, not the milder forms of HIE. It is likely that the true incidence of preterm HIE is higher than in the term population that is around 1–2/1000 live births (61).

## Clinical Spectrum

The clinical manifestations of HIE in the preterm population are vague, variable, and not well studied (Table 1). The difficulties in diagnosing HIE in preterm infants exist because the infant’s developmental immaturity adds ambiguity to differentiating clinical features (62).

**Table 1:** **Published studies on preterm HIE**.

Study (reference, type, number of patients)	Gestational age (weeks)	Criteria for HIE	Clinical features	Outcomes
Barkovich and Sargent 1995 (63), retrospective case series, *n* = 5	27–32	Profound hypoxia at birth	None mentioned	Pattern of injury on MRI
Salhab and Perlman 2005 (60), retrospective cohort, *n* = 61	31–36	Fetal acidemia (cord arterial pH <7)	Abnormal neurological examination based on Sarnat staging	3 out of 8 babies died
			Abnormal neurological outcome seen in those with low 1 and 5 min Apgar, need for CPR, mechanical ventilation	No mention on long-term neurological outcome
Logitharajah et al. 2009 (58), retrospective cohort, *n* = 55	26–36	Apgar scores <5 at 1 and <7 at 5 min	Longer duration of assisted ventilation and seizures was associated with severe outcome/death	Mainly focused on imaging pattern in preterm HI insult and long-term neurological outcome associated with imaging abnormality
		Major resuscitation (intubation/cardiopulmonary resuscitation/adrenaline) at birth		
		Brain MRI within 6 postnatal week		
		Additional inclusion criteria: abnormal intrapartum CTG, sentinel event, meconium staining, cord pH <7, neonatal seizures, and multiorgan failure		
Schmidt and Walsh 2010 (59), retrospective cohort, *n* = 12	32–36	5-min Apgar score <6	Incidence 0.9%, significant acidosis, poor tone, seizures	25% of study population died. 44% had long-term neurological adverse outcome
		Cord or initial patient blood pH <7, or base deficit >15 mmol/L		
		Evidence of encephalopathy at or shortly after birth (seizures, hypotonia)		
		History of a sentinel event at the time of delivery		
Chalak et al. 2012 (57), retrospective cohort, *n* = 9	33–35	pH <7 and base deficit >16 mmol/L	Incidence 5/1000 live births	Stages 1 and 2 had normal neurological outcomes
		Sentinel event	Graded according to Sarnat staging	
		10-min Apgar score <5, requiring assisted ventilation at birth	HIE 1 – brief ventilation, mild elevation of liver enzymes, creatinine, normal neurological examination	
			HIE 2 – multiple organ injury resolved by 7 h. Normal neuro examination at discharge. One had seizure	
			HIE 3 – severe multiorgan dysfunction, DIC, status epilepticus, prolonged ventilation, persistent abnormal neurological exam	

The neurological signs in the study by Chalak et al. used the NICHD guidelines for hypothermia and concluded that standard neurological assessment, including tone, posture, level of consciousness, spontaneous activity, primitive reflexes, and autonomic nervous system, can be applied reliably for gestational age of 33–35 weeks (64). There are no reported studies, which describe neurological signs of HIE in lower gestational (<32 weeks) age groups. The cord or initial pH was lower than 7.2 in babies with HIE in most of the studies, and persistent or delayed resolution of metabolic acidosis was associated with development of HIE (57).

## Term vs. Preterm Hypoxic–Ischemic Encephalopathy

The clinical features among term and preterm babies in HIE do overlap at many junctures, but what characteristically sets apart the preterm from term HIE are as follows:

- The higher rates of neurodevelopmental impairment as the effect of hypoxia–ischemia exaggerates vulnerability of the preterm brain.
- Preterm infants with moderate acidosis often appear well initially and often receive less intervention than term infants with the same degree of acidosis.
- Distinctive selective vulnerability of developing brain.
- Seizures in preterm infants with HIE have been reported as a marker of more severe outcome (24).

## Monitoring/Recognition of Preterm HIE

The recognition of HIE in preterm babies is difficult. Although a standard neurological examination may be applicable in the late premature group, between 33 and 35 weeks of gestation, the clinical features in the younger preterm group can be masked by physiological immaturity and, thus, can be even more challenging to diagnose. The low initial arterial pH may be a subtle marker of hypoxic ischemia and is associated with abnormal cognitive outcome in apparently low-risk preterm babies (65–68).

Preterm babies with acidotic cord or initial pH, delayed resolution of acidosis, renal impairment, raised creatinine, elevated liver enzymes, prolonged assisted ventilation, and abnormal neurological examination can be presumed to have suffered some degree of hypoxic insult. But these features may also result from etiology other than HIE (e.g., chorioamnionitis, fetal growth restriction), and patterns of enzyme changes following an asphyxial event are only described for term-born infants and older children (69, 70).

## Diagnostic Evaluation

Magnetic resonance imaging (MRI) is considered the most sensitive imaging modality for many pathological conditions of the newborn brain. Techniques, such as MR spectroscopic imaging and diffusion tensor imaging (DTI), aid in assessment of neonatal brain development. MR spectroscopic imaging measures regional brain biochemistry and is useful in assessing metabolic changes associated with brain development and injury.

### Imaging Pattern in Preterm vs. Term Brain with HIE

The classical imaging abnormalities in HIE involve three major types of lesions – PVL, basal ganglia thalami lesions, and multicystic encephalopathy. Focal non-cystic WMI is the most commonly recognized pattern of brain injury in the preterm population whereas, in term babies with HIE, two major types of injury are involved (i) *a watershed predominant pattern* involving the WM, particularly in the vascular watershed, extending to cortical gray matter following severe insult (ii) *a basal ganglia predominant pattern* involving the deep gray nuclei and perirolandic cortex, involving the cortex in severe injury (70).

In the study by Logitharajah et al. (58), WM injury was noted in around 82% of the study population and was usually diffuse and mild. Most of the infants with WM injury also had basal ganglia/thalamus injury. Infants with brain stem lesions were most likely to have incurred a severe insult. Isolated WM injury in which the cortex was spared and there was no cystic PVL was an uncommon finding, consistent with an earlier study by Barkovich and Sargent (63). The reduced susceptibility of the preterm cortex to HII may result from the lower density of a subtype of glutamate receptor allowing calcium influx into the cell leading to calcium induced excitotoxicity in the early third trimester (71, 72).

In contrast to the advantage of lesion identification, the use of MRI has a major limitation in early diagnosis since brain abnormalities might only be apparent several days after insult (73). Diffusion-weighted (DWI) MRI detects abnormalities from day 1 after birth asphyxia, but it remains unclear if the detected abnormalities relate to outcome (74, 75). However, early MRI, especially DWI, may help in detecting diffuse WM injury, which is the most dominant form of neuropathology in the premature brain (76). Given that all potential neuroprotective treatments have a limited therapeutic window of opportunity (77, 78), alternative diagnostic and predictive tools that can promptly and accurately detect abnormalities are required.

## Amplitude-Integrated Electroencephalogram/EEG

Amplitude-integrated electroencephalogram (aEEG)/electroencephalogram (EEG) are powerful tools for the diagnosis and prediction of neurological outcomes in asphyxiated infants (79, 80). Because of their practicality, and real-time interpretation, continuous monitoring with aEEG has become standard in the care of term infants with HIE (81–83). It has been suggested that the predictive accuracy of aEEG is limited compared to conventional EEG due to its data reduction and artifacts (84). However, the provision of prolonged conventional multichannel EEG with expert interpretation is not readily available in most NICUs. Thus, one or two channel aEEG is currently used more commonly in NICU (83). With respect to the assessment in preterm infants, EEGs change with uneventful maturity of the developing brain, making interpretation more complex (85–87). Specifically, the abnormal background patterns that are predictive of poor outcome at term are normal at early gestational ages. Various classification systems using conventional EEG were reported for abnormal EEGs of the preterm infants, and the findings characterized by increased discontinuity, decreased faster frequency activities, and lowered amplitudes at day 1–2 have demonstrated high predictive value of neurological outcome in infants born 27–32 weeks of gestation (88). The absence or mild depression of background activity is associated with a favorable outcome in 89%, and severe depression is associated with death in 38% and moderate or severe CP in 52% of surviving infants (85). The background pattern of aEEG 6 h after birth in asphyxiated late preterm infants born between 34 and 36 weeks of gestation has prognostic value for neurodevelopmental outcome (88). Although the data are not limited to asphyxiated infants, a recent study showed that single-channel aEEG/EEG can predict long-term outcome with 75–80% accuracy at 24 h postnatal age, even in very preterm infants born 22–30 weeks (89). In preclinical animal studies, decrease of EEG spectral edge and power are indicators of preterm brain injury following acute HI (90). Where EEG spectral edge and power were markedly and rapidly suppressed after 25–30 min HI in fetal sheep at 0.65–0.7 gestation (corresponding to 24–32 weeks gestation of brain development in human), these parameters neither recovered to baseline values within 3 days (91, 92) nor were they suppressed after 15-min HI, but did not lead to significant histopathological brain damage. These findings indicate that the changes of EEG post HI reflect the degree of injury sensitively and instantly in the preterm brain. The development of aEEG/EEG measurements and analysis may provide an effective early assessment in preterm HI injury.

## Near-Infrared Spectroscopy

Near-infrared spectroscopy (NIRS) can be useful in analyzing parameters, such as tissue oxygenation index (TOI) and regional tissue oxygen saturation (rSO_2_), of brain in a variety of pathological conditions (93). Cerebral blood flow can be indirectly measured by TOI and rSO_2_ under stable arterial oxygen saturation, and NIRS measurement of hemoglobin total (HbT) can be used to measure the cerebral blood volume (94). The abovementioned NIRS parameters may provide important information on metabolic dynamics in different brain regions and predict long-term prognosis in asphyxiated neonates (95), although this remains to be demonstrated. The utility of NIRS in evaluating oxygenation and parameters, such as blood volume, has been employed in asphyxiated term neonates (96). Tax et al. have used NIRS in assessing peripheral oxygenation in asphyxiated neonates beyond 34 weeks of gestation (97). The utility of NIRS, along with other modalities such as MRI/aEEG, has been studied in term asphyxiated neonates (98, 99). The utility of NRIS in monitoring HII in preterm neonates has not been well established.

## Auditory Brain Stem Responses

Universal neonatal hearing screening is commonly done in most NICUs. Otoacoustic emissions and automated auditory brain stem response (AABR) are standard in a number of screening programs, and infants with a history of perinatal asphyxia are overrepresented in the abnormal outcomes group (100). Generally, AABR is recommended for screening of high-risk infants. A Dutch nationwide cohort of preterm infants (born before 30 weeks gestation and less than 1000 g birth weight) who underwent AABR hearing screening, recognized severe birth asphyxia as an independent risk factor for hearing loss (101). AABRs may have a diagnostic and prognostic role to play in preterm HIE survivors.

## Neurological Outcome of Preterm HIE

There are few well-designed studies available to answer the question of neurological outcomes following preterm HIE. In the study by Chalak et al. (57), all babies with Stage 1 and Stage 2 HIE had normal neurological examinations and typical developmental milestones at 12 months of age. All surviving babies with HIE Stage 3 had poor neurological outcomes, two of nine babies died. In Logitharajah’s study (58), 15 (33%) of babies with HIE died and 15 had normal outcome, though this was not matched to severity of HIE stage. The most common type of CP observed was quadriplegic 13 (25%) followed by diplegic CP 2 (4%). A small percentage of babies had minor motor delay.

## Standard Definition

Having a standard definition of HIE in preterm infants would enable the treating physician to recognize, investigate, and prognosticate brain injury earlier during the preterm neonate’s journey. It also provides a platform base on which future studies and therapeutic interventions can be planned and implemented.

## Proposed Definition of Preterm Hypoxic–Ischemic Encephalopathy

### Definite pHIE (Both Criteria Needed)

- pH≤7 and base deficit ≥12 mmol/L in fetal/cord/first neonatal blood sample (within 1 h of birth).
- Neonatal encephalopathy – Sarnat staging [staged according to all criteria (except EEG) for infants between 33 and 35 weeks of gestation], significant changes in neurological examination and/or seizures (for infants less than 33 weeks of gestation).

### Probable pHIE (Any Two Criteria)

- pH 7.01–7.2 in fetal/cord/first neonatal blood sample.
- Early (less than 48 h) multisystem involvement, e.g., renal, liver, cardiac dysfunction.
- Preceding identifiable sentinel event (e.g., placental abruption, uterine rupture, cord prolapse) with cardiotocograph abnormalities with previously normal pattern.
- Prolonged (more than 72 h) need for assisted ventilation in absence of respiratory illness/neuromuscular disorder.
- Delayed (more than 24 h) resolution of metabolic acidosis.
- Specific region injury (predominant WM and basal ganglia injury, relative sparing of cortex) on MRI brain performed within the first week of life.

## Conclusion

Preterm HIE produces a complex, heterogeneous, and characteristic pattern of injury to the developing brain with a wide spectrum of clinical manifestations. It poses a great challenge for the treating physician to recognize, evaluate, and prognosticate. An acknowledgment and accurate definition of preterm HIE, as a distinct entity, will help in designing future studies and implementing neuroprotective strategies for treatment of this high-risk premature population.

## Author Contributions

KG drafted the initial manuscript, did the literature search, edited, and approved the final manuscript for submission. JL helped with writing section on EEG in preterm HIE, revised, and approved the manuscript for final submission. MF edited the manuscript, provided insights into the variable aspects of preterm HIE, and revised the manuscript for final submission. RH, GJ, and SM provided insights into the various aspects of preterm HIE, edited the manuscript, and approved the final submission. AM conceptualized and structured the review article, edited, and finalized the manuscript.

## Conflict of Interest Statement

The authors declare that the research was conducted in the absence of any commercial or financial relationships that could be construed as a potential conflict of interest.

## Abbreviations

AABR, automated auditory brain stem response; aEEG, amplitude-integrated EEG; BBB, blood–brain barrier; BPD, bronchopulmonary dysplasia; CP, cerebral palsy; CTG, cardiotocograph; EEG, electroencephalography; HbT, hemoglobin total; HIE, hypoxic–ischemic encephalopathy; HII, hypoxic–ischemic injury; MRI, magnetic resonance imaging; NIRS, near-infrared spectroscopy; OLs, oligodendrocytes; PVL, periventricular leukomalacia; rSO_2_, regional tissue oxygen saturation; SVZ, subventricular zone; TOI, tissue oxygenation index; WHO, World Health Organization; WM, white matter; WMI, white mater injury.
